# Giant Functional Parathyroid Cyst With “Snowstorm” Phenomenon: A Rare Clinical and Imaging Presentation

**DOI:** 10.1155/crie/9811283

**Published:** 2025-12-03

**Authors:** Ismet R. Bajrami, Brunilda I. Haxhiu, Luljeta Z. Abdullahu, Nimet H. Orqusha, Basri Z. Lenjani, Ilir N. Kurtishi, Vjollca I. Dedushaj Fazliu, Fisnik I. Kurshumliu

**Affiliations:** ^1^ Clinical Service of Nuclear Medicine, University Clinical Centre of Kosovo, Pristina, Kosovo, qkuk.org; ^2^ Faculty of Medical Sciences, Goce Delcev University, Stip, North Macedonia, ugd.edu.mk; ^3^ Clinical of Oncology, University Clinical Centre of Kosovo, Pristina, Kosovo, qkuk.org; ^4^ Faculty of Medicine, University of Prishtina “Hasan Prishtina”, Prishtina, Kosovo, uni-pr.edu; ^5^ Emergency Clinic, University Clinical Centre of Kosovo, Pristina, Kosovo, qkuk.org; ^6^ Sci. Internal Medicine-Endocrinologist, Private Praxis “Vital Health Group”, Agim Ramadani N.N., Prishtina, Kosovo; ^7^ Institute of Pathology, University Clinical Centre of Kosovo, Pristina, Kosovo, qkuk.org

**Keywords:** giant functional parathyroid cyst, magnetic resonance imaging, neck ultrasonography, snowstorm phenomenon

## Abstract

Giant functional parathyroid cysts (PCs) are extremely rare and can present significant diagnostic challenges. We report a case of a 31‐year‐old woman with primary hyperparathyroidism (PHPT) due to a giant PC, which presented with biochemical features of hyperparathyroidism, compressive symptoms in the neck structures, and a unique ultrasonographic “snowstorm” pattern, mimicking thyroid pathology. Diagnosis was confirmed postoperatively by histopathology. This case highlights the importance of including PCs in the differential diagnosis of cystic neck masses, particularly when biochemical and imaging findings are discordant.

## 1. Introduction

Parathyroid cysts (PCs) are broadly classified into functioning (producing parathyroid hormone (PTH) and associated with biochemical hyperparathyroidism) and nonfunctioning types (without hormonal activity) [1–4].

The etiology of PCs remains incompletely understood, but several mechanisms have been proposed. These include congenital origins such as vestigial remnants of the third or fourth branchial pouch or persistence of the Kürsteiner canals, coalescence of microcysts, simple retention of parathyroid secretions due to ductal obstruction, and infarction or degeneration of a preexisting parathyroid adenoma [3, 5, 6].

Most PCs are located in the neck, often near the thyroid gland or lower parathyroid glands. However, in rare cases, they may extend into the mediastinum, making diagnosis and surgical access more challenging [2, 3, 7, 8].

Functioning PCs are extremely rare and present with clinical and biochemical features of primary hyperparathyroidism (PHPT), including hypercalcemia, fatigue, nephrolithiasis, and bone disease. These cysts can produce PTH, and their cystic nature can make preoperative diagnosis difficult [3, 8–10]. Most cases are detected incidentally during imaging studies performed for unrelated reasons [11].

The majorities are non‐functional and discovered incidentally, whereas a minority are functional, presenting with clinical and biochemical features of hyperparathyroidism and representing approximately 10%–33% of all PCs described in the literature, as reported by El‐Housseini et al. [3].

First described by Sandström in 1880 and by Goris in 1905, fewer than 350 cases have been documented in the literature [2]. The condition is more frequently reported in women, with an estimated incidence ratio of 3:1 compared to men, predominantly affecting middle‐aged individuals, while pediatric cases remain rare [2]. Surgical resection is the preferred treatment for cystic parathyroid lesions and intraoperative cyst rupture should be avoided, as it may lead to misleading elevations in PTH levels. Patients with large cysts are at an increased risk of postoperative symptomatic hypocalcemia and should be closely monitored [9].

PCs are typically asymptomatic and often discovered incidentally. However, larger lesions may cause compressive symptoms or manifest as PHPT. We report a rare case of a giant functional PC with a distinctive “snowstorm” appearance on ultrasonography, coexisting with thyroid pathology and complicating the diagnostic process.

## 2. Case Presentation

### 2.1. Preoperative Findings

**Figure 1:**
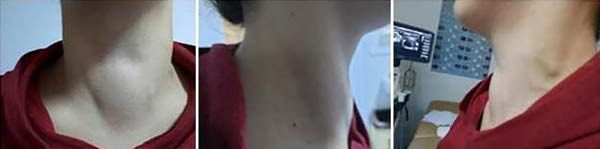
Anterior clinical view of the neck demonstrating a visible cystic swelling associated with displacement and compression of cervical blood vessels, accompanied by a palpable mass.

We present the case of a 31‐year‐old Caucasian woman of Albanian ethnicity referred for a thyroid scan due to suspected toxic multinodular goiter affecting the left thyroid lobe. She had been receiving long‐term antithyroid therapy (methimazole 2.5 mg daily). The patient reported fatigue, muscle weakness, thirst, and persistent mild pain and pressure on the right side of the neck for 8 months. Physical examination revealed a right‐sided neck swelling causing visible asymmetry (Figure 1). On palpation, a moderately complex and fluctuant mass with thrill was noted, without significant tenderness.

### 2.2. Diagnostic Assessment

At presentation, laboratory results showed thyroid function within the reference range, with free thyroxine (FT4) at 15.71 pmol/L (reference range: 9.00–20.00 pmol/L) and thyroid‐stimulating hormone (TSH) at 1.83 µIU/mL (reference range: 0.25–5.00 µIU/mL).

### 2.3. Imaging Findings

Neck ultrasound revealed a well‐defined and multilocular cystic lesion larger than 10 cm, located anterolaterally on the right side, extending beyond the right thyroid lobe into the upper mediastinum. It caused compression of cervical structures, including significant displacement and pressure on the right thyroid lobe. The lesion appeared predominantly anechoic, containing hyperechoic septa and numerous floating microcalcifications, producing a heterogeneous echotexture and a characteristic “snowstorm” appearance.

**Figure 2:**
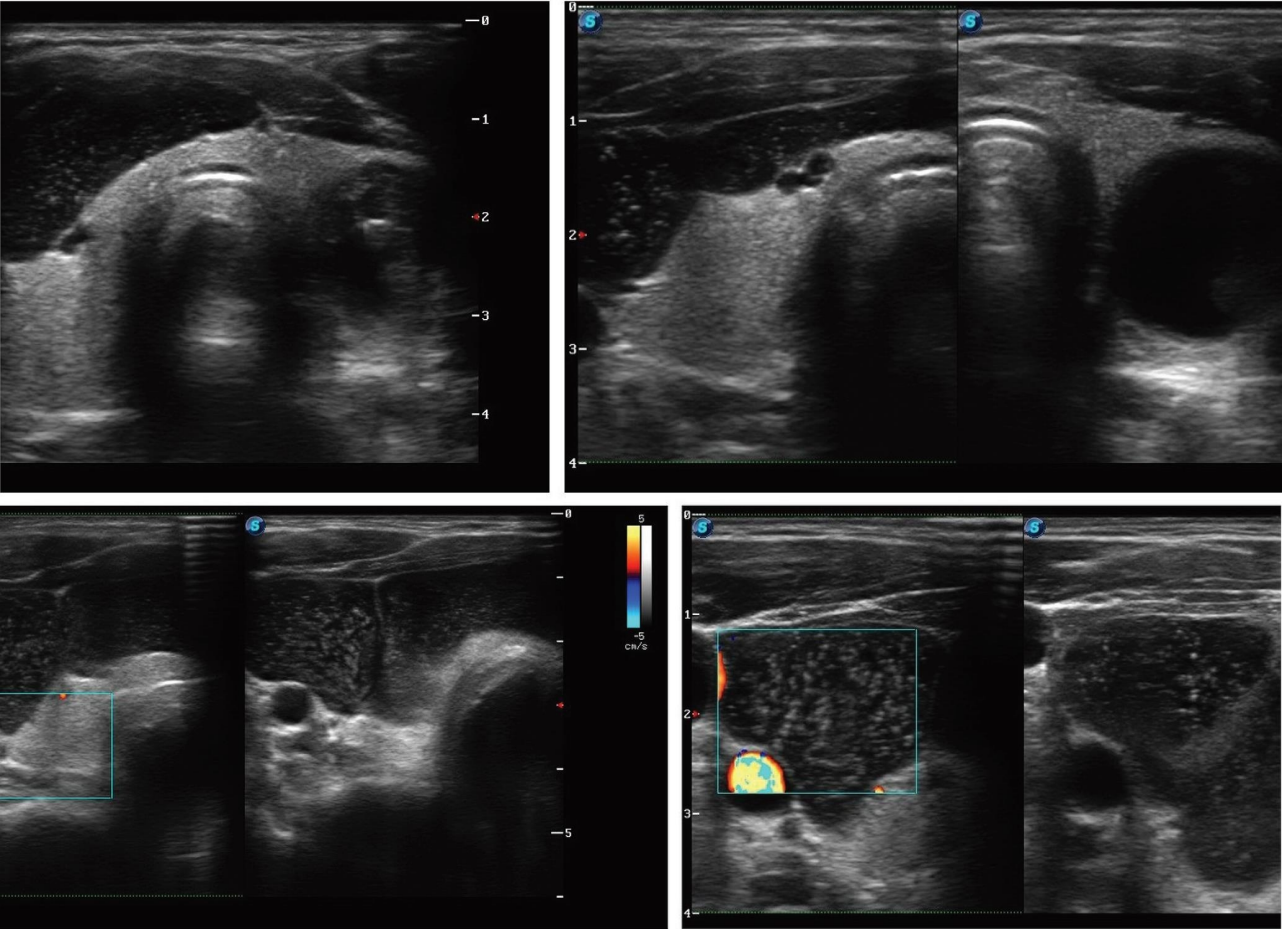
The lesion is well‐circumscribed, with internal septations and fluid content containing multiple microcalcifications, resulting in a characteristic “snowstorm phenomenon.” The left thyroid lobe shows an anechoic cystic lesion with an internal solid component, measuring 22.64 mm × 16.92 mm × 19.31 mm.

Color doppler imaging revealed no significant internal vascularity, though small vessels were observed along the septa (Figure 2).

Thyroid ultrasound showed a hypoechoic nodule (6.25 mm × 7.50 mm) in the upper medial third and iso‐ to hypoechoic nodules in the lower pole of the right lobe. The left lobe demonstrated an anechoic cystic lesion (22.64 mm × 16.92 mm × 19.31 mm) and a hypervascular and isoechoic adenomatous nodule (20.60 mm × 16.70 mm × 17.80 mm).

**Figure 3:**
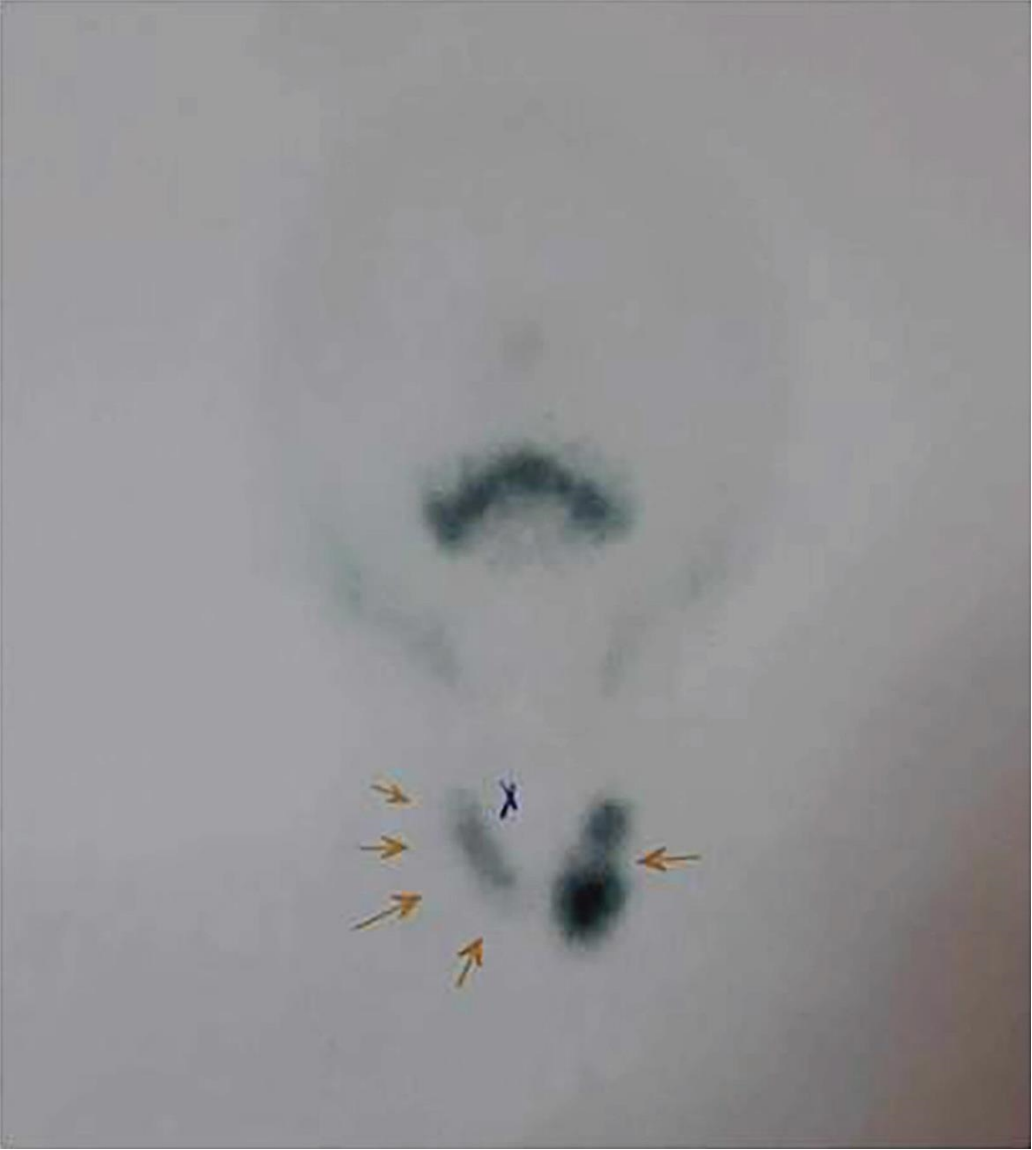
Thyroid scintigraphy with 99mTc‐pertechnetate showing suppression of the right lobe compressed by the adjacent parathyroid cyst, with a cold nodule in its upper third. The left lobe demonstrated a cold cystic nodule in the upper pole and a hot adenomatous lesion in the lower pole.

Technetium‐99m pertechnetate scintigraphy revealed a cold and nonfunctioning area at the cyst site, confirming its extrathyroidal origin. The right lobe appeared compressed and suppressed, with a cold nodule in its upper medial third. In contrast, the left lobe showed a cold cystic nodule in the upper third and a hot and hyperfunctioning adenomatous lesion in the lower pole (Figure 3).

**Figure 4:**
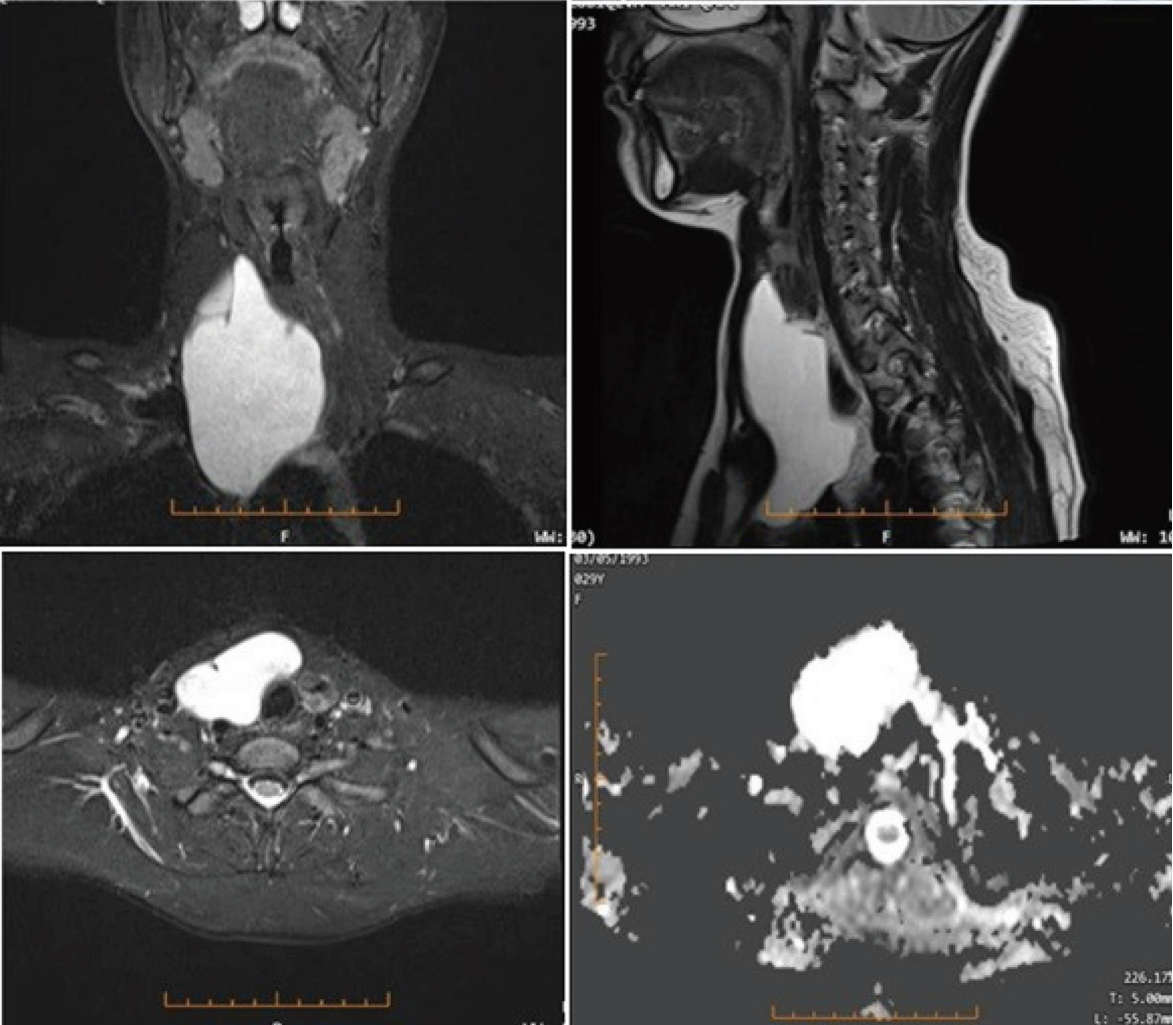
Magnetic resonance imaging (MRI) showing a well‐circumscribed, septated, T2‐hyperintense cystic lesion in the anterior right cervical region with extension into the upper anterior mediastinum. The lesion displaced the right thyroid lobe posteriorly and caused mild tracheal deviation without stenosis. It measured approximately 3.8 cm × 7 cm × 10 cm, contained proteinaceous fluid and showed no contrast enhancement.

The patient declined a 99mTc‐sestamibi (MIBI) scan but consented to magnetic resonance imaging (MRI). Functional PCs may lack Tc‐99m sestamibi uptake, complicating preoperative localization. MRI revealed a well‐circumscribed T2‐hyperintense cystic lesion with internal septations, extending from the right cervical region into the upper anterior mediastinum. It was located between the right thyroid lobe and superficial neck muscles, causing posterior displacement of the thyroid and slight tracheal deviation to the left, without evidence of stenosis. The cyst measured 3.8 cm × 7 cm axially and 10 cm craniocaudally, showed high protein content, and demonstrated no contrast enhancement. It was in contact with the brachiocephalic veins and superior vena cava, without signs of invasion or thrombosis. An additional 2 cm × 2 cm adenomatous lesion was identified in the left thyroid lobe (Figure 4). The parotid and submandibular glands, parapharyngeal spaces, and pharynx appeared normal, with no pathological lymphadenopathy.

Based on the ultrasound findings, additional lab tests were ordered, revealing PTH of 298 pg/mL (31.5 pmol/L; reference range: 9.0–94.0 pg/mL or 0.95–10.0 pmol/L), ionized calcium of 1.36 mmol/L, and 25‐hydroxyvitamin D deficiency (below 10 ng/mL). These findings supported the diagnosis of PHPT (Table 1).

**Table 1:** Preoperative laboratory tests.

Parameter	Patient value (SI)	Reference range (SI)	Patient value (conventional)	Reference range (conventional)
ALP	258 U/L	<240 U/L	258 U/L	<240 U/L
Acid phosphatase	3.30 U/L	<5.3 U/L	3.30 U/L	<5.3 U/L
Total calcium	2.68 mmol/L	2.15–2.57 mmol/L	10.74 mg/dL	8.8–10.4 mg/dL
Ionized calcium	1.41 mmol/L	1.10–1.40 mmol/L	5.64 mg/dL	4.4–5.6 mg/dL
Phosphorus	0.90 mmol/L	0.87–1.45 mmol/L	2.79 mg/dL	2.7–4.5 mg/dL
PTH	298 pg/mL	9–94 pg/mL	298 pg/mL	9–94 pg/mL
25‐Hydroxyvitamin D	9.5 ng/mL	30–70 ng/mL	9.5 ng/mL	30–70 ng/mL
Urea	7.8 mmol/L	1.7–8.3 mmol/L	21.8 mg/dL	4.8–23 mg/dL
Creatinine	102.5 µmol/L	44–80 (F)/53–106 (M)	1.16 mg/dL	0.50–0.90 (F)/0.70–1.20 (M)
FT4	18.71 pmol/L	9.0–20.0 pmol/L	1.45 ng/dL	0.70–1.55 ng/dL
TSH	1.83 µIU/mL	0.25–5.00 µIU/mL	1.83 µIU/mL	0.25–5.00 µIU/mL

## 3. Treatment

### 3.1. Surgery and Histopathology

**Figure 5:**
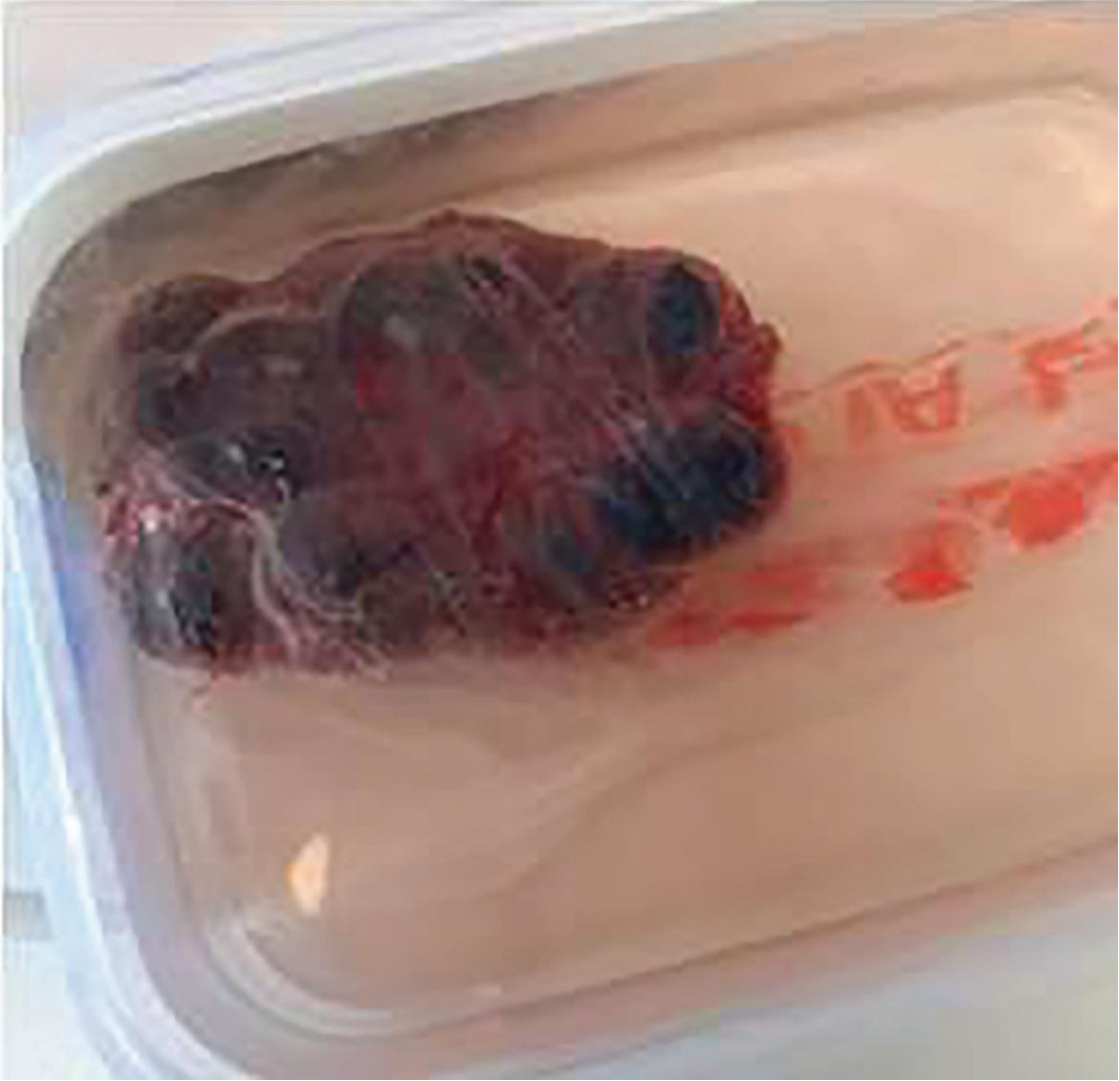
Macroscopic view of the excised parathyroid cyst following parathyroidectomy.

To exclude malignancy and assess potential adhesions to surrounding structures, the patient underwent an anterotransverse cervicotomy with complete excision of the cystic mass (Figure 5).

Intraoperatively, the cyst was found adherent to the posterior surface of the right thyroid lobe and extended inferiorly into the upper mediastinum. The lesion was well‐encapsulated, with thin fibrotic walls and no gross invasion of adjacent structures. The recurrent laryngeal nerve and inferior thyroid vessels were carefully preserved. Approximately 20 mL of brownish cystic fluid was aspirated before excision to facilitate dissection. No evidence of local infiltration or enlarged lymph nodes was observed. The excised specimen measured 9.0 cm × 5.5 cm × 3.5 cm, with a smooth external surface and a unilocular cystic cavity containing residual brownish fluid. A thin rim of adjacent thyroid tissue was noted adherent to one portion of the cyst wall, consistent with intraoperative adhesions to the posterior aspect of the right thyroid lobe.

Microscopic examination revealed a cyst wall composed of dense fibrotic tissue lined by flattened to cuboidal epithelial cells and monomorphic cells with eosinophilic, granular (oncocytic) cytoplasm arranged perivascularly—features often observed in parathyroid adenomas. The cystic lumen contained small cystic spaces, hemosiderin‐laden macrophages (siderophages), and proteinaceous material.

Immunohistochemical staining demonstrated strong nuclear positivity for GATA3 (clone L50‐823) and negativity for TTF‐1 (clone 8G7G3/1), confirming the parathyroid origin of the lesion. These findings were consistent with a cystic parathyroid adenoma. (Histopathology and immunohistochemistry figures were not available for inclusion due to technical limitations.)

### 3.2. Outcome and Follow‐Up

Following surgery, levels of PTH and calcium returned to normal. The patient was monitored for 18 months with regular endocrinological follow‐up, during which no recurrence or complications were observed (Table 2). Throughout this period, the patient continued antithyroid therapy with Methimazole 2.5 mg daily to maintain stable thyroid function, with clinical and laboratory parameters remaining within normal limits.

**Table 2:** Laboratory tests after operative treatment.

Parameter	Patient value (SI)	Reference range (SI)	Patient value (conventional)	Reference range (conventional)
ALP	188 U/L	<240 U/L	188 U/L	<240 U/L
Acid phosphatase	3.00 U/L	<5.3 U/L	3.00 U/L	<5.3 U/L
Total calcium	2.37 mmol/L	2.15–2.57 mmol/L	9.48 mg/dL	8.8–10.4 mg/dL
Ionized calcium	1.34 mmol/L	1.10–1.40 mmol/L	5.36 mg/dL	4.4–5.6 mg/dL
Phosphorus	0.97 mmol/L	0.87–1.45 mmol/L	3.0 mg/dL	2.7–4.5 mg/dL
PTH	78 pg/mL	9–94 pg/mL	78 pg/mL	9–94 pg/mL
25‐Hydroxyvitamin D	39.5 ng/mL	30–70 ng/mL	39.5 ng/mL	30–70 ng/mL
Urea	6.7 mmol/L	1.7–8.3 mmol/L	18.8 mg/dL	4.8–23 mg/dL
Creatinine	98.25 µmol/L	44–80 (F)/53–106 (M)	1.11 mg/dL	0.50–0.90 (F)/0.70–1.20 (M)
FT4	17.71 pmol/L	9.0–20.0 pmol/L	1.38 ng/dL	0.70–1.55 ng/dL
TSH	0.89 µIU/mL	0.25–5.00 µIU/mL	0.89 µIU/mL	0.25–5.00 µIU/mL

*Note:* Conversion factors—calcium (mmol/L × 4.0 = mg/dL); phosphorus (mmol/L × 3.1 = mg/dL); creatinine (µmol/L × 0.0113 = mg/dL); urea (mmol/L × 2.8 = mg/dL); FT4 (pmol/L × 0.0777 = ng/dL). Reference ranges adjusted for sex where applicable.

## 4. Discussion

### 4.1. Incidence and Clinical Presentation

PCs are rare in endocrine surgery and clinical practice, accounting for 0.8%–3.41% of all parathyroid lesions and affecting 0.075% of the general population [4]. Most cystic parathyroid adenomas are located in the neck, although approximately 10% are found in the mediastinum [10]. In our case, the parathyroid adenoma was located primarily in the neck with partial extension into the upper mediastinum.

### 4.2. Cystic Fluid Characteristics

Optic clear aspirate fluid is often thought to suggest a PC; however, fluid characteristics are variable. In our case, a 10‐cm cyst with thin walls contained brown fluid. This is consistent with recent reports, including Rajasekaran et al. [12], who described a nonfunctioning PC with brownish fluid in a large cyst. Also, the study “Cystic Parathyroid Lesions: Functional and Nonfunctional Parathyroid Cysts” (JAMA Surgery) noted that while many PCs yield clear and colorless fluid, turbid or colored fluid aspirate does not exclude the diagnosis of a PC [9. Therefore, fluid color alone should not be used to rule out PC diagnosis.

### 4.3. Functional Nature and Biochemical Findings

The functional PC in our patient was discovered during evaluation for thyroid disease due to toxic multinodular goiter. Initially, the patient was receiving thyrostatic therapy, with a TSH level of 0.02 µIU/mL. At the time of examination, thyroid hormone levels had normalized, thyroglobulin (Tg) was elevated at 108 ng/mL, and PTH was significantly increased at 298 pg/mL (reference range: 9.0–94.0 pg/mL).

Measurement of Tg and PTH levels in cystic aspirates is an important diagnostic step for differentiating PCs from thyroid cysts and other cystic neck lesions. However, in our case, these measurements were not performed, and the diagnosis was established solely through histopathological examination [3, 13].

### 4.4. Role of Imaging in Diagnosis

Ultrasound is a rapid, cost‐effective, and widely available imaging technique for detecting neck masses and assessing their local extent [14, 15]. It provides valuable information on size, shape, structure, and internal composition, often revealing multilocularity with septa and calcifications. In our case, ultrasound demonstrated a distinctive “snowstorm” phenomenon a feature frequently described in hydatid cysts and popliteal cysts, but rarely reported in PCs. Expanded discussion: This sonographic finding has only rarely been reported in PCs; possible mechanisms include microcalcifications or proteinaceous colloid material within the cystic content.

This sonographic appearance may be attributed to internal calcified content or particulate material within the cystic lesion. Although seldom documented in parathyroid lesions, similar patterns have been observed in cystic or necrotic components of other neck masses. Such an appearance can serve as an important imaging clue, suggesting the presence of calcifications and aiding in the differentiation of PCs from other cystic neck pathologies (Hazhan S. Ultrasound of the Thyroid and Parathyroid Glands; LiVolsi VA, Baloch ZW. Cystic lesions of the neck: Cytologic and radiologic correlation. Arch Pathol Lab Med. 2000).

Cystic parathyroid adenomas with cystic and microcalcified components due to cystic degeneration have been previously described by Rogers et al. [16] and later confirmed in several recent reports [17. Additionally, atypical parathyroid adenomas may mimic parathyroid carcinoma, necessitating meticulous histopathologic evaluation to assess for vascular and capsular invasion to distinguish between these entities [18].

### 4.5. MRI and Differential Diagnosis

Although PCs are typically less vascular, their fluid content produces a high signal on T2‐weighted MRI sequences, which facilitates their detection [15]. In our case, MRI findings initially suggested a lymphangioma. However, differentiating PCs from cystic thyroid nodules with calcifications or benign and malignant cystic tumors was critical for determining the appropriate management strategy.

### 4.6. Management and Surgical Considerations

Although many PCs are incidental and asymptomatic, larger cysts—particularly those extending into the mediastinum can cause local compression effects such as mechanical airway obstruction or vascular displacement [6]. In such cases, surgical intervention is warranted.

While airway compression was not evident in our patient, the decision to perform surgery was multifactorial. The surgical decision was based on several factors: the lesion’s large size (>6 cm), high PTH levels, diagnostic uncertainty, and lack of response to aspiration. Furthermore, giant functional PCs have been rarely associated with parathyroid carcinoma, although infrequent. While no universal consensus exists, most endocrine surgical guidelines recommend excision of functional or symptomatic PCs, especially if larger than 3 cm, unresponsive to aspiration, or with diagnostic uncertainty. According to the 2016 American Association of Endocrine Surgeons (AAES) guidelines for PHPT, surgery is recommended in symptomatic individuals or in asymptomatic patients with marked hypercalcemia, osteoporosis, nephrolithiasis, or reduced renal function. Our patient presented with persistent hypercalcemia, significantly elevated PTH levels, and a giant cyst of uncertain originall valid indications for surgical excision.

Our patient did not have osteopenia but had mild hypercalciuria, with no evidence of nephrolithiasis or renal impairment.

Malignancy in PCs is exceedingly rare, accounting for less than 1% of all PHPT cases. However, larger cystic lesions may mimic malignancy and raise concern due to their size and functional profile.

The cyst was completely excised via an anterotransverse cervicotomy, resulting in prompt symptom relief without postoperative hypocalcemia. This favorable outcome underscores the efficacy of early surgical management in symptomatic cases. PCs should be carefully distinguished from other cystic neck lesions, including thyroid cysts, thyroglossal duct cysts, and branchial cleft cysts. Fine‐needle aspiration biopsy (FNAB) should be performed with extreme caution because of the potential risk of cyst rupture or intralesional hemorrhage, which may result in cervical hematoma and, in rare cases, acute hypercalcemic crisis. Therefore, a thorough multimodal diagnostic approach is warranted, particularly in patients with elevated serum PTH levels [2, 4, 5, 9]. Despite the patient’s refusal to undergo MIBI scintigraphy and FNAB, preoperative biochemical markers combined with ultrasound findings provided sufficient diagnostic confidence [3, 7, 9]. Even though cystic parathyroid adenoma is a rare diagnosis, it should be considered in the differential diagnosis of neck masses and measurement of PTH in aspirated cyst fluid is highly recommended. In our case, cyst fluid aspiration for PTH analysis was not performed; however, the markedly elevated serum PTH levels and postoperative histopathological findings confirmed the diagnosis. The large size and hyperfunctioning nature of the cyst, together with associated biochemical abnormalities, warranted surgical removal. Elevated PTH levels in the cystic fluid confirmed the parathyroid origin of the lesion. Nonfunctioning PCs with compressive symptoms or esthetic concerns also require surgical treatment. Functioning PCs are managed with surgical resection, while mediastinal location, uncertain diagnosis, or suspicions of malignancy represent additional indications for surgery [2, 3, 5, 6, 8]. Preoperative evaluation of bone health (DEXA) and kidney function was normal. Even in the absence of airway compromise, the cyst’s size and potential risks of sudden growth or rupture supported the decision for surgery.

## 5. Conclusion

This case highlights the need to consider PCs in the differential diagnosis of cervical cystic masses, particularly when thyroid dysfunction and elevated PTH levels are present. Although rare, cystic parathyroid adenomas can be a cause of PHPT, warranting thorough evaluation.

Biochemical analysis of serum and, when possible, cyst fluid, along with imaging modalities such as ultrasound and MRI, are essential for accurate diagnosis and surgical planning. Surgical excision remains the treatment of choice, especially for symptomatic or large lesions. This case reinforces the value of a multimodal diagnostic approach in ensuring timely and effective management of these uncommon but clinically important entities.

### 5.1. Learning Points

• Giant functioning PCs, although rare, should be considered in the differential diagnosis of anterior neck masses.
• Specific ultrasound features, such as the “snowstorm” appearance, may provide important diagnostic clues suggestive of parathyroid origin.

A comprehensive approach combining biochemical analysis and multimodal imaging is essential to differentiate PCs from other cystic neck lesions and to guide appropriate treatment.

## Ethics Statement

This case report does not involve interventional experiments. It is a retrospective presentation of a rare clinical case, based on patient records, biochemical findings, and imaging assessments. All diagnostic and therapeutic procedures were conducted in accordance with established clinical guidelines and ethical standards. This study does not involve experiments on animals.

## Consent

Written informed consent was obtained from the patient for the use of medical information and associated images for academic and publication purposes. All data have been anonymized to ensure patient privacy and confidentiality.

## Disclosure

This manuscript has not been previously published nor has been submitted in preprint form on any public platform. The authors have not self‐archived the article prior to its submission to this journal. All authors meet the criteria for authorship as defined by the International Committee of Medical Journal Editors (ICMJE) and have approved the final version of the manuscript and agreed on the respective responsibilities. All clinical, biochemical, and imaging data are authentic and accurately reflect the case and authors’ professional assessments. No data manipulation, plagiarism, or scientific misconduct has occurred.

## Conflicts of Interest

The authors declare no conflicts of interest.

## Author Contributions

Ismet R. Bajrami initiated the case report and approved the final version of the manuscript. Brunilda I. Haxhiu oversaw manuscript preparation, coordinated submission, ensured compliance with ethical standards, and approved the final content. Luljeta Z. Abdullahu drafted the original manuscript, assisted in surgical data documentation, and was actively involved in data acquisition and interpretation. Nimet H. Orqusha processed and curated all imaging figures and contributed significantly to the literature review, reference formatting, and editing of the procedural description. Ilir N. Kurtishi participated in data interpretation and provided critical revision for intellectual content. Basri Z. Lenjani managed clinical follow‐up, performed relevant procedures, and contributed to therapeutic planning. Vjollca I. Dedushaj Fazliu provided valuable clinical insight and collegial support during the diagnostic and therapeutic decision‐making process. Fisnik I. Kurshumliu contributed to histopathological analysis and interpretation. Ismet R. Bajrami and Luljeta Z. Abdullahu contributed equally to this work and share first authorship.

## Funding

No external funding was received for the preparation of this case report.

## Supporting Information

Additional supporting information can be found online in the Supporting Information section.

## Supporting information


**Supporting Information** Dynamic ultrasonographic demonstrate a “snowstorm” phenomenon with mobile echogenic particle within the cyst. The dynamic “snowastorm” appearance is shown in Supporting Video.

## Data Availability

All original data generated or analyzed during the preparation of this case report are included in this published article.
